# Variations in Soft and Hard Tissues following Immediate Implant Placement versus Delayed Implant Placement following Socket Preservation in the Maxillary Esthetic Region: A Randomized Controlled Clinical Trial

**DOI:** 10.1155/2021/5641185

**Published:** 2021-10-04

**Authors:** Muthukumar Santhanakrishnan, Nithyakalyani Ramesh, R. Kamaleeshwari, Vedavalli Subramanian

**Affiliations:** ^1^Faculty of Dental Sciences, Department of Periodontology, Sri Ramachandra Institute of Higher Education and Research, Porur, Chennai, 600116 Tamil Nadu, India; ^2^Department of Periodontology, Sri Ramachandra Institute of Higher Education and Research, India

## Abstract

**Introduction:**

Although retrospective analysis has shown immediate placement of implants (IIP) in the maxillary esthetic zone showing promising outcomes compared to delayed placement of implants following socket preservation (DIP), a direct comparison in a prospective, well-designed randomized fashion with adequate power analysis between the two implant placement protocols is still lacking. This study is aimed at radiographically evaluating the effect of IIP after extraction as compared to implant placed in preserved sockets 4 months following extraction (DIP) in terms of changes in buccal plate thickness(CBT) after 6 months of healing and evaluation of pink esthetic score (PES) for assessment of soft tissue changes and patient-related outcome measures (PROMs) using visual analogue scale (VAS).

**Materials and Methods:**

25 implants were placed immediately following extraction in the IIP group, and 25 implants were placed four months following socket preservation with demineralized bovine bone mineral (DBBM) and advanced platelet-rich fibrin (A-PRF) in the DIP group, control group, in the maxillary esthetic region. CBCT was taken preoperatively and 6 months postoperatively to assess the dimensional changes in the buccal bone plates(CBT). PES and PROMs for pain threshold and patient satisfaction using VAS were evaluated at the time of implant placement and 6 months postoperatively.

**Results:**

Significant differences in mean reduction in buccal plate thickness (CBT) were found in the test group (IIP) 0.2 ± 0.02 compared to the control group (DIP) which showed a mean reduction in CBT of 0.4 ± 0.1 (*p* < 0.001) at the end of 6 months. Although there was no statistically significant difference in PES between the groups, there was a significant difference between the groups when individual values of PES were compared at *p* < 0.001.

**Conclusion:**

The IIP group showed lesser reduction in CBT and a better PES which is an important clinical information which could be translated clinically in situations where implant placement is planned in the maxillary esthetic region. This trial is registered with CTRI/2019/06/019723.

## 1. Introduction

Extraction of tooth causes resorption of bundle bone leading to significant changes in soft and hard tissues surrounding the socket eventually leading to reduction in both horizontal and vertical directions, which cannot be prevented with the help of the techniques which are currently available. Conventionally, implants are placed after the sockets are healed to reduce the complications following implant placement. Alveolar ridge preservation (ARP) has been done to reduce the degree of bone resorption and to improve functional and esthetic outcomes.

Several materials have been successfully used for ARP, which are basically made of matrix scaffolding materials and biologic agents. Matrix scaffolding materials are osteoconductive and act as space maintainers providing dimensional stability. Among these wide choices of materials, DBBM has been popularly used for ARP. However, the limitation in using bovine bone includes its delayed rate of resorption and healing with fibrous encapsulation with limited or no remodelling of the augmented socket towards its central aspect [1, 2].

Biologic agents are molecular mediators with osteoinductive properties and facilitate de novo bone formation [3]. Matrix scaffolding materials and biologic agents have been used together to achieve optimal surgical outcome [4]. Among the available biomaterials, PRF has been used commonly in regenerative periodontal therapy as it promotes healing of hard and soft tissues [5–7] by releasing growth factors over a prolonged period from the autologous bioscaffold of dense fibrin matrix by regulating cellular events which facilitate mitosis, proliferation of osteoblast, vascularization, and collagen synthesis.

Currently, A-PRF, a modified version of conventional PRF which releases higher concentration of growth factors prepared by utilising lower G-forces, has shown optimal regenerative potential in healing extraction sites [8, 9]. The addition of platelet concentrates facilitates to establish a conducive environment for bone regeneration by creating a synergy between the action of growth factors and the attraction of target cells facilitated by osteoconductive scaffolds. However, ARP procedure prolongs the time needed for implant placement by 3-6 months and also requires a second surgical procedure for implant insertion [10, 11].

In recent years, the demand for immediate implant placement (IIP) has considerably increased especially in the anterior region for esthetic reasons. Immediate implants are placed immediately after extraction of the teeth in a fresh extraction socket. An important advantage of IIP is that it facilitates improved final esthetic outcome by decreasing the amount of bone resorption that naturally follows tooth extraction. One of the main advantages of IIP is the reduced treatment time as there is no necessity to wait for soft and hard tissues to heal as in early and delayed implant placement protocols; however, IIP is associated with greater risk of failure and complications. However, when the jumping distance (space between the implant and buccal bone) is grafted, it reduces the horizontal changes of hard and soft tissues by approximately 0.5 mm after IIP [12].

However, the superiority of IIP compared to delayed implant placement (DIP) following socket preservation has not been established so far as there are only limited human studies comparing both these techniques in the maxillary esthetic region. Moreover, patient-reported outcomes (PROs) are now regarded as a fundamental measure of therapeutic success [13, 14]. However, current literature reveals only a limited number of studies reporting on patient-centered outcomes in addition to objective evaluations of implant-supported rehabilitations in the anterior maxilla.

Hence, the present study is aimed at comparing clinically and radiographically the dimensional alterations in soft and hard tissues following immediate implant placement and delayed implant placement following socket preservation. The primary outcome was the assessment of horizontal dimensional changes of the buccal plate of bone. The secondary outcome included pink esthetic score (PES) evaluation and assessment of pain threshold and patient satisfaction using visual analogue scale (VAS).

## 2. Materials and Methods

### 2.1. Study Design

This study was a prospective controlled, randomized, clinical investigation according to the CONSORT statement (http://www.consortstatement.org/). All procedures and materials were approved by the institutional ethical committee (Ref. IEC/19/APR/150/20) and monitored following the Good Clinical Practice. The trial was registered at CTRI http://www.clinicaltrials.gov/ (Ref. CTRI/2019/06/019723).

### 2.2. Sample Size

To calculate the number of patients to be treated, summary statistics (mean and standard deviation) reported by Sun et al. [15] was used for the variable labial bone thickness, respectively, for the IIP group and the DIP group. The effect size was equal to 1.1, and this value was used to determine the sample size based on a two-independent sample Mann–Whitney test (two-tailed) with a significance level alpha set equal to 5% and power equal to 95%. G-power software, V.3.1, was used. This resulted in 19 subjects for each group.

### 2.3. Population

Participants were selected on a consecutive basis among patients of the Outpatient Department of Periodontology and Implantology, Sri Ramachandra Dental College and Hospital, Chennai, between May 2019 and May 2020. The patients agreed to participate in the study by signing a written informed consent, in full accordance with the ethical principles of the Declaration of Helsinki on experimentation involving human subjects, as revised in 2008.

### 2.4. Inclusion Criteria

- Patients in the age group of 18–50 years were selected
- Missing maxillary teeth from premolar to premolar with healthy adjacent teeth bilaterally
- Intact socket following tooth extraction (type I) and a labial bone thickness of ≤2 mm in CBCT
- Intact facial alveolar bone wall without soft tissue or bone defects

### 2.5. Exclusion Criteria

- History of systemic disease
- Recent infectious diseases or surgical treatment within 30 days
- Smokers
- Pregnancy or lactation
- Patients on regular medications affecting periodontal healing (e.g., phenytoin, dihydropyridines, calcium antagonists, and cyclosporine) or anticoagulant therapy with warfarin, clopidogrel, ticlopidine, and aspirin
- Presence of pathological lesions around the surgical area
- Perforated labial cortical plate seen in CBCT
- Patients undergoing radiation therapy or history of radiation within the last two years
- Patients with a history of psychiatric illness or allergy to the drugs or anesthetics under evaluation
- Uncooperative patients and patients who were not willing to participate in the study to report for follow-up

## 3. Preoperative Evaluation

Preoperative assessment of clinical parameters was performed which included assessment of oral hygiene using Oral Hygiene Index-Simplified (Greene and Vermillion 1964), determination of tissue biotype—thick or thin (Muller 2000), pocket probing depth using a UNC-15 periodontal probe (Hu-Friedy Mfg. Co., Chicago, IL, USA), and soft tissue esthetic assessment using the pink esthetic score (PES) [16].

## 4. CBCT Standardisation

Evaluation of labial cortical thickness at the below-mentioned levels was done using Ray Scan Alpha Plus (LED Medical Diagnostics Inc.) cone beam 3D imaging system with high resolution 70 *μ*m voxel, standard exposure time 14 (s), tube voltage 90 kVp and 10 Ma, and the FOV (field of view) was collimated to 5 cm by 5 cm to limit the radiation exposure. Data were acquired as a volume acquisition and reconstructed in multiple planes.

In order to recreate the same reference points preoperatively before extraction and postextraction following implant placement, a fixed reference point (*r*) was created by drawing a line from the coronal most aspect of the labial alveolar crest (*r*_2_) paralleling with the long axis of the inner aspect (oral aspect) of the labial alveolar crest or the facial aspect of the tooth (preoperatively) and implant (postoperatively) following the natural inclination of the tooth/implant extending apically so as to bisect the floor of the maxillary sinus or the nasal fossa (*r*_1_) [17].

The sagittal views were plotted to measure the bone dimensional changes as follows.

**Figure 1:**
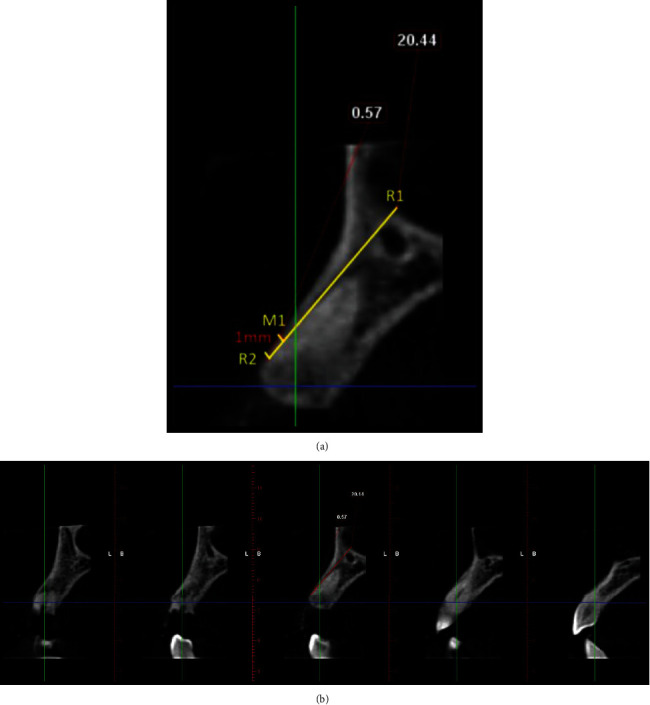
Demonstration of a treated case: radiographic measurements. (a) Preoperative CBCT: sagittal view. (b) Labial cortical thickness assessed using sections of 1 mm thickness.

To study the labial cortical thickness in cross sections, 1 mm sections were used and the thickness of the labial cortical plate was measured 1 mm from the most coronal aspect of labial bone crest using the distance measurement tool in labiopalatal direction preoperatively (*m*_1_) (Figure 1).

### 4.1. Randomization Process and Allocation Concealment

**Figure 2:**
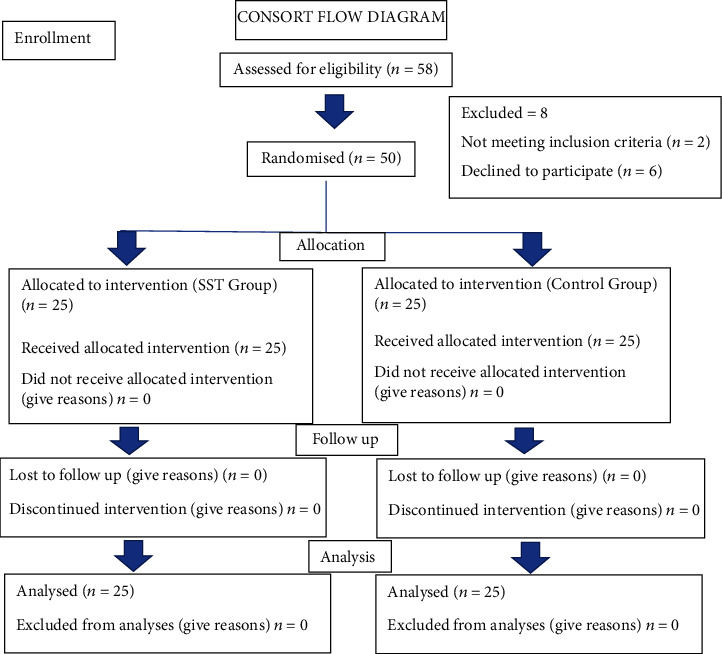
Consort flow diagram: *n* representing the implant sites which is considered the population.

Randomization was performed using a computer-generated list by someone not involved in other aspects of the study. Allocation concealment was performed by opaque continuously numbered sealed envelopes that were opened after tooth extraction and assessment of the integrity of the bone plates (Figure 2).

### 4.2. Treatment Procedures

**Figure 3:**
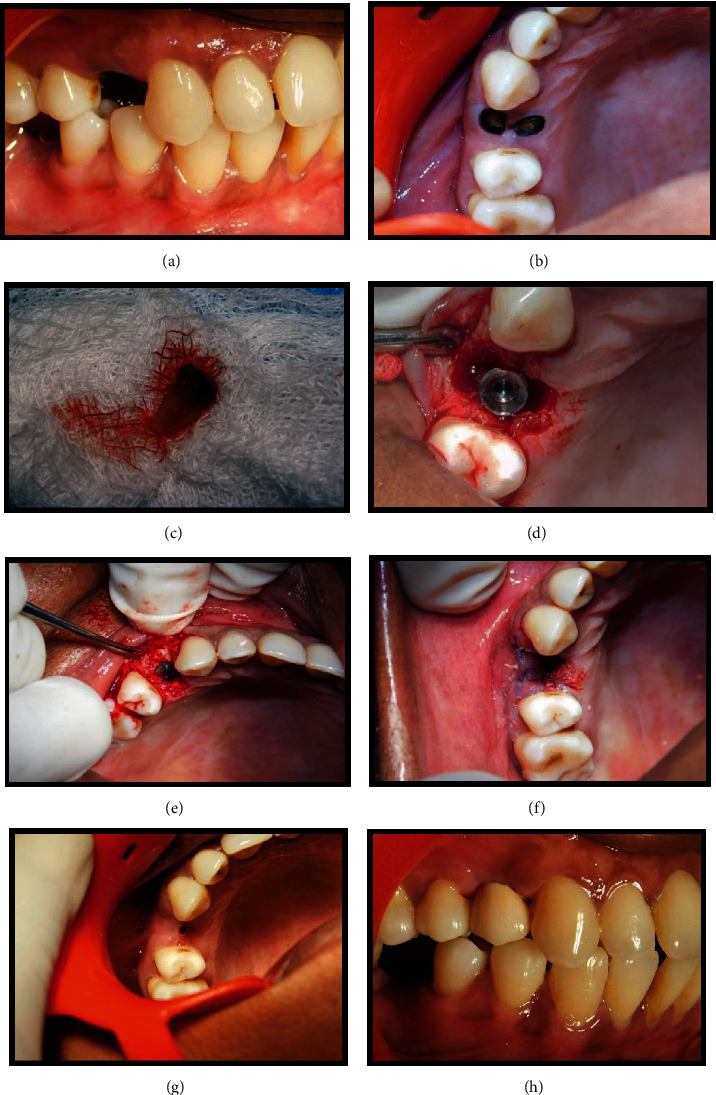
Immediate implant placement in relation to 14 (test group). (a) Pre-operative site in relation to 14. (b) Root stumps present in relation to 14. (c) Extracted root stumps (14). (d) Implant placed in relation to 14. (e) Jumping distance grafted using DBBM. (f) Immediate postoperative view. (g) Four-month post-operative view. (h) Metal-ceramic crown placed in relation to 14.

A full-thickness envelope flap including the mesial and distal tooth was performed, and the tooth was extracted with great care to preserve the buccal bone plate and the surrounding hard tissue (Figures 3(a)–3(c)). Granulation tissue was carefully removed with hand instruments, and sterile saline rinses were performed. After assessment of the integrity of the bone plates, patients were randomly assigned to the following groups.

#### 4.2.1. Test Group (IIP)

There was immediate implant placement, plus a deproteinized bovine bone mineral (DBBM) and autogenous bone grafted into the gap up to the buccal bone crest.

#### 4.2.2. Control Group (DIP)

A combination of deproteinized bovine bone mineral (DBBM) and advanced plate-rich fibrin (A-PRF) in a 1 : 1 ratio was grafted into the socket up to the buccal bone crest, sealed with a partially epithelized connective tissue graft which was harvested from the hard palate. Implants were placed four months following socket preservation.

In detail, in the study group (IIP), an atraumatic extraction using periotome and forceps was performed to preserve the available alveolar bone, and then, the socket was debrided gently following tooth extraction using curettes and irrigated by physiologic saline solution. The osteotomy was directed in a palatal position leaving a gap of approximately 2 mm between the implant and the labial plate (Figure 3(d)). The implant was placed 2 to 3 mm apical to the bone crest [18].

The space between the implant and the alveolar socket wall (test group, IIP) was grafted using autogenous bone particles (obtained during osteotomy) and xenograft (DBBM; Bio-Oss, Geistlich) in a 1 : 1 ratio (Figure 3(e)). Small flaps, about 3 to 4 mm long, were elevated. A provisional restoration with a 2 mm gap between the restoration and the surgical site was maintained for soft tissue to fill in both the groups. The provisional crowns were then constructed chairside on stock straight titanium abutment with an emergence profile to support the coronal tissues. Provisional crowns were not in contact with the opposite dentition, both in the static and dynamic occlusion.

**Figure 4:**
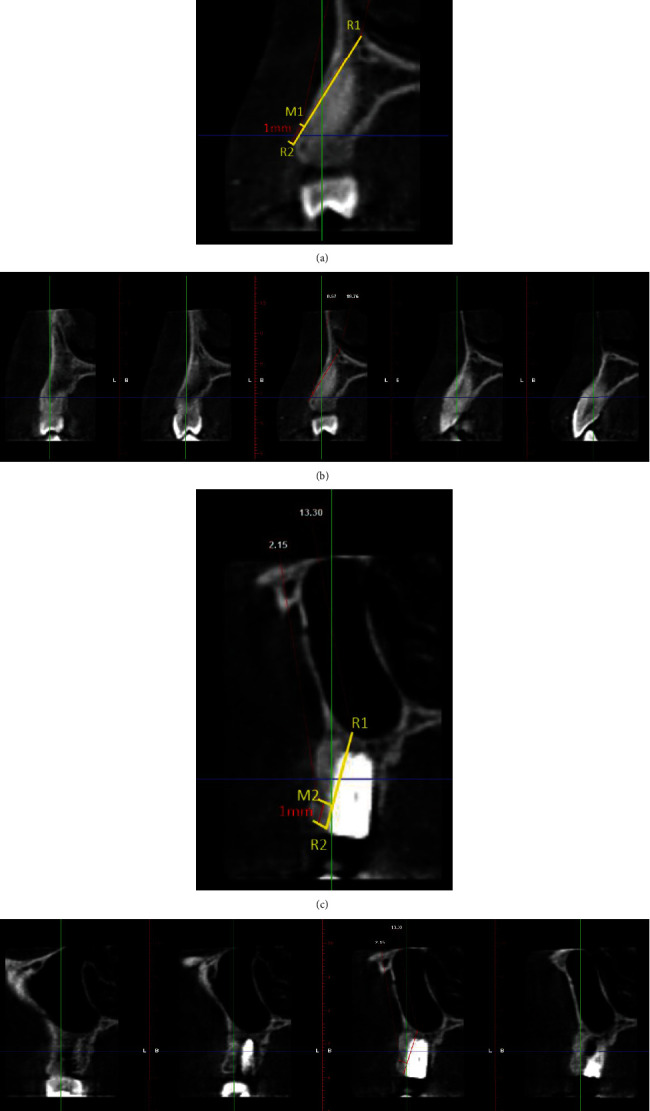
Radiographic evaluation of immediate implant placement in relation to 14 (test group). (a) Pre-operative CBCT: sagittal view. (b) Labial cortical thickness assessed using sections of 1 mm thickness. (c) Post-operative CBCT: sagittal view. (d) Labial cortical thickness assessed using sections of 1 mm thickness.

Four months after tooth extraction, patients of the immediate group had the implants assessed for stability, impressions were taken at implant level, using copy transfer and individualised trays, and metal-ceramic crowns were fabricated and provisionally cemented on customised titanium abutments within 2 weeks, after the impressions were taken (Figure 3(h)). To evaluate the changes in the labial cortical thickness, CBCT images were taken preoperatively and 6 months postoperatively (Figures 4(a)–4(d)) and the measurements were made as described previously.

#### 4.2.3. In the Control Group

**Figure 5:**
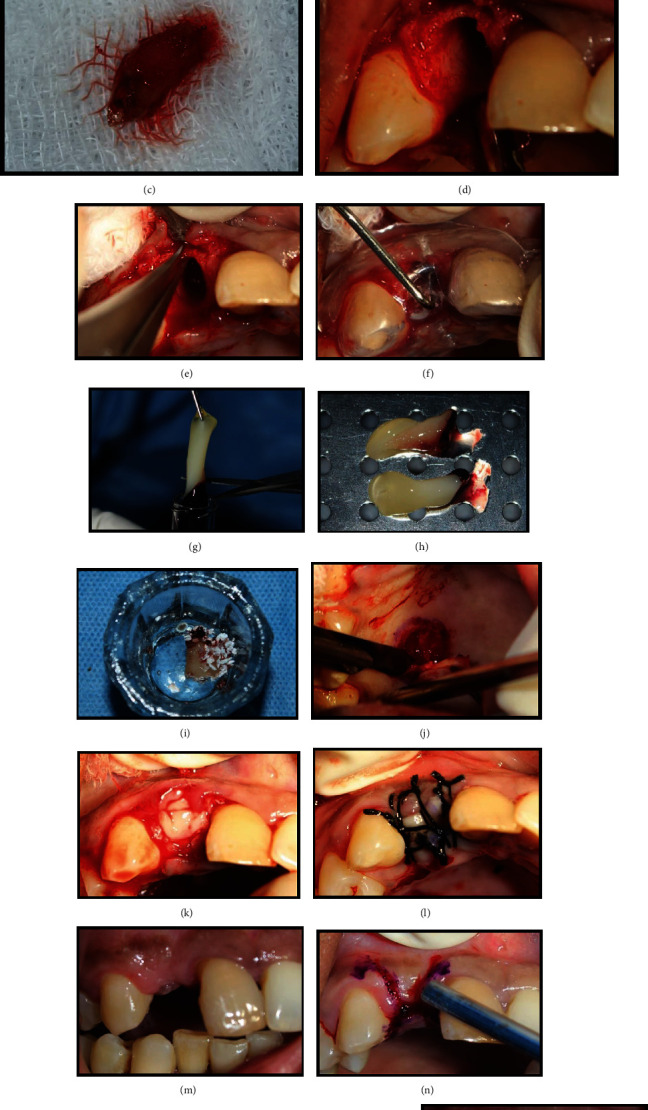
Delayed implant placement in relation to 12 (control group). (a) Pre-operative site in relation to 12. (b) Atraumatic extraction of 12. (c) Extracted root stumps (12). (d) Socket in relation to 12 postextraction. (e) Clinical measurement of labial plate thickness. (f) Measurements of the socket using a stent. (g, h) A-PRF procured from the patient's own blood. (i) A-PRF and DBBM mixed in a ratio of 1 : 1. (j) Free gingival graft procured from the palate. (k) Socket seal achieved using FGG following placement of A-PRF and DBBM in the extraction socket. (l) Sutures placed in relation to 12. (m) Post-operative site 4 months following preservation. (n) Outline of incisions for implant placement in relation to 12. (o) Placement of implant in relation to 12. (p) Placement of healing abutment in relation to 12. (q) Crown placed in relation to 12.

An atraumatic extraction using periotomes and forceps was performed to preserve the available alveolar bone, and then, the socket was debrided gently following tooth extraction using curettes and irrigated by physiologic saline solution (Figures 5(a)–5(c)). Morphology of the extracted socket was recorded through direct measurements using indexed stents and UNC-15 periodontal probe (Hu-Friedy, Chicago, USA) (Figures 5(d)–5(f)). Venous blood was collected via venipuncture of the forearm in the antecubital vein into a 10 ml sterile glass vacuum tube by a trained phlebologist. The blood sample was immediately centrifuged at 1300 rpm (200 × *g*) for 14 minutes. The A-PRF clot was separated from the three distinct layers that formed within the tube [9].

The A-PRF clot was cut into small pieces, and DBBM was added to achieve a final volume with a 1 : 1 ratio of graft particulate (DBBM; Bio-Oss, Geistlich) to A-PRF (Figures 5(g)–5(i)). The socket was filled with this mixture up to the bony crest with light compression. For wound closure and soft tissue augmentation, a partially epithelized connective tissue graft was harvested from the hard palate (Figure 5(j)). After the graft was harvested, the complete donor area was sutured using a transverse mattress suture [19]. The graft was then carefully inserted into the socket using a guiding suture and placed over the socket orifice (Figures 5(k) and 5(l)). Follow-up evaluations were done at periodic intervals. Oral hygiene instructions were reinforced throughout the study period. At 4 months postsurgery, the test sites were reentered for implant placement (Figure 5(m)).

Patients of the delayed group had implants placed after similar procedures were completed, as previously described, for the immediate group. After local anaesthesia flaps were elevated, implant sites were prepared without cleaning the preserved socket, and implants were placed and provisional non-occluding acrylic crowns were cemented within 24 h. Three months after implant placement, patients of the delayed group had the implants assessed for stability and permanent restorations were given as described for the test group (Figures 5(n)–5(q)).

**Figure 6:**
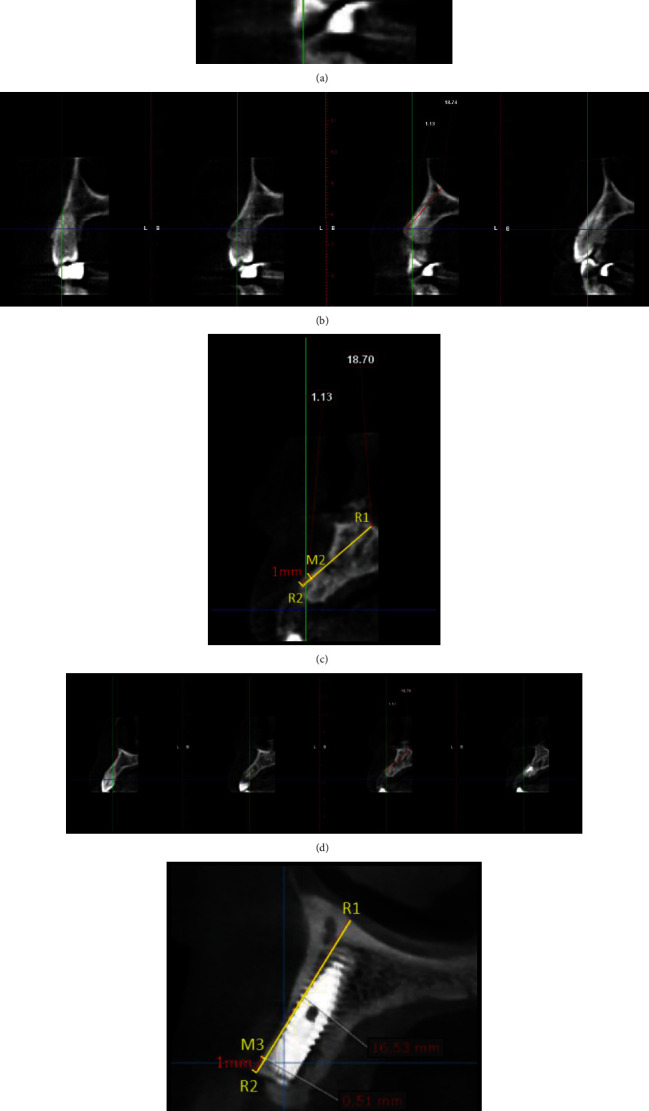
Radiographic evaluation of delayed implant placement in relation to 12 (control group). (a) Pre-operative CBCT: sagittal view. (b) Labial cortical thickness assessed using sections of 1 mm thickness. (c) Post-operative CBCT: sagittal view. (d) Labial cortical thickness assessed using sections of 1 mm thickness. (e) Post-implant CBCT: sagittal view. (f) Labial cortical thickness assessed using sections of 1 mm thickness.

Postoperative medications included an oral antibiotic, a dose of 500 mg thrice daily for 5 days (amoxicillin with lactobacillus (Novamox LB Cap., Cipla Ltd., India)) and an oral analgesic, a dose of 400 mg three times daily for 5 days (ibuprofen (Imol tab. Zydus Cadila HealthCare Ltd.)). The patients followed strict oral hygiene measures and regular rinsing of chlorhexidine 0.2% (Clohex-ADS, Dr. Reddys Laboratories, India) mouthwash for 2 weeks. The patients were followed up every other day for the first week and then every ten days for the first month and 4 months postoperatively. To evaluate the changes in the labial cortical thickness, CBCT images were taken preoperatively and 6 months postoperatively (Figures 6(a)–6(f)) and the measurements were made as described previously.

### 4.3. Statistical Analysis

Statistical analysis was performed using the SPSS software package (version 20.0; SPSS Inc.). The numerical data were explored for normality by checking the distribution of data and using tests of normality (Shapiro Wilk test). The changes in labial bone thickness showed normal (parametric) distribution, while PES data showed non-parametric distribution.

The parametric data were expressed as mean ± SD, and non-parametric data was expressed as median (IQR). For parametric data, one-way ANOVA was used, and for comparison between the groups and within group, differences were tested by using repeated measures ANOVA. For non-parametric data, Wilcoxon signed-rank test was used for within-group comparison and Mann–Whitney “*U*” test was used for between-group comparison.

## 5. Results

### 5.1. General Information

The study population consisted 58 subjects that were screened for participating in this clinical trial from May 2019 to July 2019. Of these patients, 6 patients were excluded as they were not willing to comply with the follow-up visits and two patients were excluded due to the loss of buccal plate after tooth extraction. A total of 50 subjects (24 men and 26 women) were finally recruited, randomized, and included in the clinical trial. 25 were allocated to the IIP group (test), and 25 were allocated to the DIP group (control) (Table 1).

### 5.2. Clinical Outcomes

#### 5.2.1. Radiographic Outcomes

The radiographic interpretation was emphasized to measure the changes in the buccal cortical thickness one mm apical to the most coronal bone crest for both the groups.

**Figure 7:**
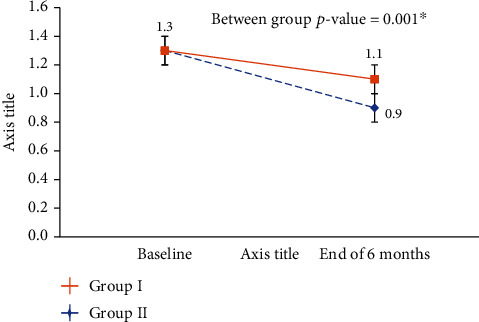
Change in CBT of Group I vs. Group II of the study participants (*n* = 50). ^∗^*p* < 0.05 considered as significant using one-way ANOVA for between-group and repeated measures ANOVA for time points.

Dimensional changes in the buccal plate at 6 months after placement from the baseline were assessed. However, patients in the test group (IIP) had significantly higher values of CBT compared with the control group (DIP) at 6 months (*p* < 0.001) (Table 2, Figure 7).

**Figure 8:**
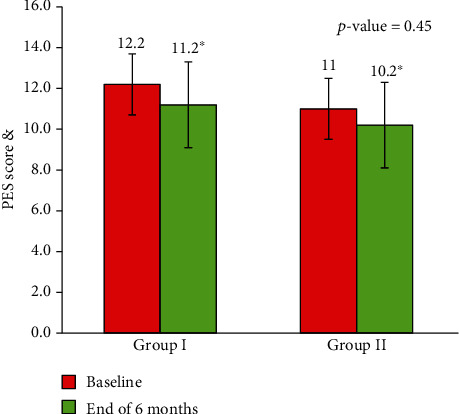
Change in PES score between Group I and Group II of the study participants (*n* = 50).

The PES was assessed by two trained blinded clinicians. Based on PES, there were no significant differences in the groups at 6 months after implant placement from the baseline. The patients in the IIP group had a preoperative PES value of 12.2 ± 1.9, which was not significantly different from the control group 10.9 ± 1.5 (*p* = 0.07). At 6 months, PES in the SST group was slightly higher than that in the control group, with no statistical significance (11.7 ± 1.8 versus 11.2 ± 2.1, *p* = 0.07). The proportion of patients with individual PES values was grouped into three categories (score < 10, score > 11 < 12, and score > 13 and <14) and was analysed for changes between the groups. There was a significant difference in individual PES values between the groups with higher proportion of patients in the IIP group demonstrating PES values of score > 13&14 which was highly statistically significant at *p* < 0.001. Patients in the control group had significantly lower values of PES scores (score > 13 and <14) compared with those in the IIP group at 6 months (*p* < 0.001), indicating better esthetic outcome in the SST group (Table 3, Figure 8). The intraclass correlation coefficient test was done to test the interevaluator consistency which showed excellent agreement (*p* = 0.83 for the IIP group and *p* = 0.88 for the control group) (Table 4).

#### 5.2.2. Patient-Centered Outcome

VAS score for pain and esthetics showed no statistically significant difference between the groups at *p* = 0.72 and *p* = 0.48, respectively. However, the trend showed a better esthetic satisfaction in the IIP group (64% of scores 9 and 10) compared to the DIP group (52%) (Tables 5 and 6).

## 6. Discussion

This trial was done to evaluate whether immediate implant placement following extraction of the tooth would be beneficial or delayed placement of implant after tooth extraction and following socket preservation would be beneficial in the maxillary esthetic region. Evidences from literature clearly showed that only a very limited number of randomized clinical trials compared immediate and delayed implants; moreover, no prospective controlled studies are available comparing immediate implant placement and delayed placement of implants following socket preservation [20–23]. Hence, the purpose of this prospective RCT was to evaluate and compare immediate versus delayed implant placement following socket preservation in terms of the volume loss of bone (CBT) and esthetic evaluation (PES) around single dental implants in the maxillary esthetic region.

One of the key factors in the success of implant therapy, especially in the maxillary esthetic region is patient satisfaction; hence, a visual analogue scale was used to analyse patient's satisfaction for esthetics and pain. A VAS value of 7-8 has been described to represent good esthetic results, while higher values 9-10 indicate optimum implant esthetics [14, 24]. Case selection was carefully done limiting to include teeth which are non-restorable, and patients with type 1 extraction sockets alone were included to ensure intact bony wall at the time of implant placement to prevent any variations in assessment of CBT that could occur if patients with varying degrees of resorption of the buccal bony wall were included.

In the test group, implants were placed so as to create a direct contact between the bone and implant in the apical region and to create a marginal gap buccally in the coronal most portion. Hence, IIP resulted in engaging 3-4 mm of bone apical to the socket. The implants were intentionally placed lingually to ensure that 2/3 of the implant surface comes in contact with the lingual wall of the socket of the extracted tooth thereby facilitating good primary stability and ensuring the success of implant therapy. However, the jumping distance created between the implant and the tooth socket buccally was grafted by using a combination of autogenous bone particles (obtained during osteotomy) and xenograft (DBBM 1 to 2 mm; Bio-Oss, Geistlich) in a 1 : 1 ratio.

Bovine bone granules were used as due to their low substitution rate they preserve and support the bundle bone apart from acting as a scaffold providing an osteoconductive surface by holding the clot and facilitating proliferation of capillaries and homing of growth factors and stem cells. However, autogenous bone was also added which was derived during the preparation of the osteotomy site for implant placement to enhance the osteoinductive property for faster bone formation.

Bone substitutes have been widely employed in dental practice for socket preservation, as they help in maintaining the ridge dimension, but there are some important factors to be considered in choosing the optimal bone graft like the degree of bioresorbability which is detrimental in new bone formation and also the appropriate time when the prosthetic rehabilitation is needed [25].

A slowly resorbing graft material can be beneficial by providing good space maintenance throughout the entire time course of healing [26–29]. Hence, DBBM was used in the control group (DIP) of the present study as the slow substitution rate of DBBM coincides with the peak of osteoblastic activity after extraction, i.e., after 8 weeks. However, they are usually associated with remnant graft material and reduced amount of vital bone at the time of implant insertion. Hence, to facilitate vital bone formation, A-PRF was added to DBBM as it releases intrinsic and concentrated growth factors at the appropriate time during healing which ensures vital bone formation. The socket entrance was sealed using a partially epithelized connective tissue graft harvested from the hard palate for wound closure and soft tissue augmentation.

Flaps with minimal extension were elevated to facilitate better access in both the groups. The interproximal crestal bone loss is of practical importance, and its loss has been found to be less following the use of a limited flap design versus the widely mobilized flap procedure. Hence, paramarginal incisions were used to elevate a flap with minimal extension and gain access so as to preserve the attached gingival width and papilla was not elevated interproximally so as to prevent papillary loss and prevent any impairment in the esthetic results [30]. The primary outcome variable in the present study was changes in the crestal bone thickness (CBT) as it determines the integrity of soft and hard tissues around the implant. There was a highly statistically significant difference (*p* = 0.001) in crestal bone thickness from baseline to 6 months between the groups (for the test group, it was 0.2 ± 0.02 and 0.4 ± 0.1 for the control group). Even though the mean difference was only 0.2 mm, it presents a lot of clinical relevance as the buccal wall thickness is <1 mm in the maxillary esthetic region [31] and it will make a huge difference in the soft tissue alterations as observed in our study. The insertion of bovine bone granules and autogenous bone in the gap between the implant and the bony socket wall could have successfully prevented resorption of the buccal bundle bone in the IIP group [14, 24]. However, there was an increased reduction in the thickness of buccal bone in the DIP group as a result of remodelling resorption that would have occurred in the four-month interval between socket preservation and implant placement. Even if randomized trials describing the result from analysis of crestal bone thickness are not available for some treatment comparisons, that is, in particular between immediate versus delayed placement of implants, the results concerning linear and horizontal remodelling have echoed the trend already seen in the present study regarding bone loss in the esthetic zone.

The difference in reduction of buccal plate thickness between the groups in the present study was 0.20 ± 0.1 which was significantly greater than values of other studies where they showed a difference of 0.07 Felice et al. 2016 [22], 0.06 Esposito et al. 2015 [32], and 0.05 Felice et al. 2015 [23]. The difference could be due to the fact that patients with varying degrees of bone loss with 4 mm of buccal wall missing were included as compared to type 1 sockets with intact bony walls included in the present study. Moreover, in the study by Felice et al. in 2016 [21] and 2015 [20] in the delayed implant sites, alveolar ridge preservation was not done which could have resulted in greater bone loss in the delayed implant group.

The results of the present study were in accordance with Battista et al. [33], where in a retrospective study for 3 years in 9 IIP and 10 DIP patients, lower bone volume bone loss (less than 10%) was observed in the IIP group compared with the loss registered for implants placed with the DIP group with a loss of 24% of the initial volume of interest. The percentage of bone loss in the delayed group (27.1%) showed two times the percentage of bone loss in the immediate group (14.6%). This clearly confirmed that immediate placement and restoration of a single implant was a successful option of treatment in the case of single compromised teeth, as attested by previous authors with good results in terms of short/long-term success (close to 100%) and marginal bone loss (from 0.42 to 2.69 mm) [34, 35]. Moreover, immediate placement of dental implant protocol seems to maintain the preexisting architecture of soft and hard tissues in most cases, as reported in the literature [36].

The results of the present study also concurred with Pellicer-Chover et al. [37], where they showed that although there was no statistically significant difference in linear bone loss the delayed implant placement group demonstrated a greater volume of bone loss 0.66 + 0.25 compared to 0.54 + 0.39 mm in the immediate implant group. They also showed that the loss of crestal bone volume around an implant-supported crown appeared higher in the DIP (control group) than the IIP (test group). The results were more predictable for the IIP following restoration. However, they included anterior as well as posterior teeth in their study and involved fixed full-arch prosthesis as compared to single tooth restorations in the maxillary esthetic region in the present study.

The results of the present study for the IIP group differed from a study by Clementini et al. [38], where they treated 10 patients with IIP and grafted the jumping distance with DBBM and collagen membrane and showed a difference in buccal bone changes of 0.99 ± 0.21 mm four months following the procedure, and Sanz et al. [12] in a study on 43 patients in anterior maxilla observed that when IIP was done along with grafting the jumping distance with DBBM followed up to 4 months and demonstrated reduction of 1.1 mm (29%) in the buccal plate thickness compared to 1.6 mm (38%) when the jumping distance was not grafted, and they emphasized the need to graft the jumping distance irrespective of the defect size. The present study showed a difference of 0.2 ± 0.02 compared to the above studies, which could be attributed to the use of autogenous bone in addition to DBBM for grafting the jumping distance, and a reduced follow-up time of four months in their study.

In a study by Block et al. [39] in 2009, they compared immediate and delayed implants in terms of changes in vertical and horizontal bone levels and changes in facial gingival margin in 55 patients in maxillary anterior and premolar teeth with a 2-year follow-up and concluded that there was no significant difference in changes in the bone levels 0.55 mm vertically and 3.03 ± 0.98 mm horizontally in the immediate group and 0.46 mm vertically and 2.91 ± 0.87 mm horizontally in the delayed group. They concluded that the changes in the crestal bone levels will be determined by depth and configuration of the implant irrespective of whether it is placed immediately are delayed following socket preservation. However, the results were not comparable with the present study as the inclusion criteria were not specific and they used only digital periapical radiographs as compared to CBCT to assess bone level changes in the present study.

In a study by Palatella et al. [40] in 2008, they compared immediate implants and delayed implants placed 8 weeks after extraction in 16 patients involving maxillary anteriors and premolars and found a mean marginal bone resorption of 0.54 ± 0.51 mm in the immediate group and 0.46 ± 0.54 in the delayed group after 2 years of implant placement. They attributed the bony changes to the implant design which had a 1.8 mm transmucosal smooth collar which resulted in the rough smooth interface of the implant being placed 3.8 mm from the CEJ of the adjacent teeth as the implants were placed 2 mm below the CEJ of the adjacent teeth facilitating bone resorption in both the groups. The delayed group showed greater resorption as socket preservation was not done. Moreover, nonstandardised periapical radiographs were used to assess bone level changes which could have confounded the results of the study. Hence, the results of this study could not be compared with the present study.

Earlier implant therapy was focused merely on osseointegration and optimal function, but currently, esthetics holds an integral part in the success of implant therapy especially in the anterior maxilla. Hence, an objective evaluation of esthetic outcome becomes essential for the success of implant therapy. Therefore, PES [16] was used as secondary outcome variable to determine the soft tissue changes in both groups in the present study.

Although there was no statistically significant difference in PES between the groups at 6 months post-implant placement, the trend clearly showed better PES values in the IIP group at 6 months post-loading. The average of PES scores that were assessed by two blind assessors was 11.2 ± 2.1 in the test group compared to 10.2 ± 1.4 in the control group, and there was a highly statistically significant difference in individual PES values between the groups with higher proportion of patients (40%) in the IIP group demonstrating PES values of score > 13&14 compared to the DIP group which had significantly lower values (4%) of PES scores (score > 13 and <14), indicating better esthetic outcomes in the IIP group which could be attributed to lesser bone resorption observed in the IIP group compared to the DIP group.

In a study by Block et al. in 2009 [39], t as described early demonstrated a significantly coronally positioned gingival margin in the IIP group compared to the DIP group which resulted due to the recession of facial gingival margin by 1 mm in the delayed group which occurred during the healing phase following socket preservation which did not happen in the IIP group as the gingival margin was supported by the crown. However, they noticed that following crown placement there was no further changes in the position of gingival margin in the DIP group. This probably could be the reasoning behind the better PES scores observed in the IIP group in the present study.

The results of our study were comparable to the results of Felice et al. [23] who demonstrated in 50 patients (25 in the IIP group and 25 in the DIP group) after one year of loading, the average PES score, assessed by a blind assessor, to be better for the immediate group(PES score of 12.78 ± 1.09) compared to the delayed group(PES score of 12.22 ± 1.13) at 12 months after loading although it was not statistically significant.

The results of our study for PES were comparable to a study by Nadaff Pour et al. [41] in 2018 where they treated 42 patients, 22 in the IIP group and 18 in the DIP group, in which implants were placed 6 months following extraction and demonstrated a better PES score (a mean PES score of 8.54 ± 1.26 in the IIP group compared to 8.10 ± 1.65 in the delayed group). They attributed the loss of alveolar prominence in both groups for the inferior PES score observed in their study.

The results of our study for PES differed from the study of Tonetti et al. [42] in 2016 where they compared IIP and DIP 12 weeks after extraction in 124 patients (62 in each group) with 1-year follow-up. They demonstrated inadequate PES in 19% of the DIP group compared to 42% in the IIP group. They attributed the increased surgical risk in the IIP group to have impacted the final soft tissue esthetics. However, they included patients with varying degrees of bone loss in their study which could have impacted the results.

The superior PES score of the IIP in the present study could be due to the immediate restoration with a provisional crown in the present study as demonstrated in a study by Arora et al. in 2018 where they examined 40 patients (20 patients of IIP with immediate provisional and 20 patients of IIP with delayed restoration placed 3-4 months following implant placement) and concluded that the timing of restoration impacted the esthetic outcome as demonstrated by their results where IIP with immediate restoration showed a mean PES of 11.1 + 2.08 and IIP with delayed restoration showed 10.3 + 2.23.

However, current review of the literature reveals only a limited number of studies reporting on patient-centered outcomes in addition to objective evaluations of implant-supported rehabilitations in the maxillary esthetic region. Hence, the patient-centered outcome measures for pain and esthetic satisfaction were analysed in the present study using a visual analogue scale. Although there was no significant difference between the groups for both parameters, the trend clearly showed a better esthetic satisfaction in the test group with 64% of the patients demonstrating scores of 9 and 10 compared to the control group (52%) which could be attributed to maintenance of the pre-existing architecture of soft and hard tissues and a lesser number of visits in the test group compared to the control group. The results of our study differed from a study by Tonetti et al. where they compared IIP and DIP in 124 patients (62 in each group), evaluated PROMs by using a VAS for pain and esthetics, and demonstrated no significant difference between the groups with >85% esthetic satisfaction in both groups and a moderate pain and discomfort in both groups (VAS < 3). However, the inclusion parameters were wider in their study involving patients with varying degrees of loss of the buccal alveolar bone and socket preservation was not done in the DIP group. Hence, the results were not comparable with our study.

The results of the present study revealed no significant difference between the groups with regard to PROMs. However, there was a lesser reduction in buccal plate thickness and correspondingly lesser soft tissue changes when implants were placed immediately rather than waiting for implant placement following socket preservation. The present study demonstrates that there is a significant reduction in CBT and PES irrespective of whether the implants were placed immediately following extraction (IIP) and 4 months following socket preservation. However, the IIP group showed lesser reduction in CBT and a better PES which is an important clinical information which could be practiced clinically in situations where implant placement is planned in the maxillary esthetic region.

Nevertheless, the present study had a short follow-up period of 6 months and smaller sample size and inclusion of cases was strictly restricted to situations were the buccal socket wall was intact. Future multicentric studies should be conducted with longer follow-up periods and broader inclusion criteria with varying levels of buccal bone loss and increased sample size, which would give a definitive indication of timing of implant placement following extraction in the maxillary esthetic zone.

## Figures and Tables

**Table 1:** Demographic variables.

Variables^$$^	Group I	Group II	*p* value^∗^
Age (years)	29.8 ± 9.7	30.8 ± 6.5	0.97
Gender			
Male	12 (48%)	11.0 (44%)	0.7
Female	13 (52%)	14 (56%)	0.7
CBT^$^	1.3 ± 0.1	1.3 ± 0.1	0.05
PES	13 (2.0)	13 (2.0)	0.62

^∗^ *p* < 0.05 considered as significant using Kruskal-Wallis test for continuous and chi-square test for categorical variable. ^$^*p* value was tested by using one-way ANOVA. ^&^Average of observer I and observer II. ^$^Parametric data was expressed as mean and SD. ^$$^Nonparametric data was expressed as median (IQR).

**Table 2:** Comparison of labial bone thickness in the studied groups at different time intervals (*n* = 50).

Variables^$$^	Group I (*n* = 25)			Group II (*n* = 25)			Effect size (d)	*p* value^∗^
	Baseline	End of 6 months	Difference^^^	Baseline	End of 6 months	Difference^^^		
CBT^$^	1.3 ± 0.1	1.1 ± 0.1	0.2 ± 0.02^∗^	1.3 ± 0.1	0.9 ± 0.1	0.4 ± 0.1^∗^	2.8	**0.001**

^∗^ *p* < 0.05 considered as significant using one-way ANOVA for between-group comparisons and repeated measures ANOVA for within-group comparisons.

**Table 3:** Changes in PES score of the study participants (*n* = 50).

Variables	Group I (*n* = 25)			Group II (*n* = 25)			*p* value
	Baseline	End of 6 months	Difference^^^	Baseline	End of 6 months	Difference^^^	
PES^&^	12.2 ± 1.9	11.2 ± 2.1	1.0 ± 1.0	10.9 ± 1.5	10.2 ± 1.4	0.7 ± 1.0	0.45^∗^
Median (IQR)	13.0 (2.0)	12.0 (4.0)	1.0 (1.0)^#^	11.0 (1.5)	10.5 (1.5)	1.0 (1.0)^#^	
Score ≤ 10.0 *n* (%)	3.0 (12.0)	8.0 (32.0)	5.0	6.0 (12.0)	11.0 (44.0)	5.0	**0.002** ^$^
Score ≥ 11 and ≤12 *n* (%)	6.0 (24.0)	11.0 (44.0)	1.0	16.0 (64.0)	13.0 (52.0)	-3.0	
Score ≥ 13 and ≤14 *n* (%)	16.0 (64.0)	10.0 (40.0)	-6.0	3.0 (12.0)	1.0 (4.0)	-2.0	

^#^ *p* < 0.05 considered as significant using the Wilcoxon signed-rank test. ^∗^*p* < 0.05 was tested by using the Mann–Whitney *U* test. ^$^*p* < 0.05 considered as significant using Fisher's exact test.

**Table 4:** Test and retest reliability score of PES of the study participants (*n* = 50).

	Observer 1				Observer 2			
	Baseline	End of 6 months	Difference	Baseline	End of 6 months	Difference	Effect size (d)	Agreement value
Group I (*n* = 25)	12.4 ± 1.9	11.3 ± 2.2	1.0 ± 1.0	12.0 ± 1.9	11.2 ± 2.0	0.8 ± 1.2	0.5	0.83
Group II (*n* = 25)	11.0 ± 1.6	10.4 ± 1.5	0.6 ± 1.0	10.8 ± 1.4	10.0 ± 1.3	0.8 ± 1.1	0.0	0.88

<0.40: poor agreement; 0.40–0.59: fair agreement; 0.60–0.74: good agreement; 0.75–1: excellent.

**Table 5:** VAS score for pain between Group I and Group II of the study participants (*n* = 50).

Variables	Group I (*n* = 25)	Group II (*n* = 25)	*p* value
VAS score			
Score 0, *n* (%)	5.0 (20.0)	5.0 (20.0)	0.72
Score 1, *n* (%)	17.0 (68.0)	19.0 (76.0)	
Score 2, *n* (%)	3.0 (12.0)	1.0 (4.0)	
Score 3, *n* (%)	5.0 (20.0)		

^∗^ *p* value was tested by using Fisher's exact test.

**Table 6:** VAS score for esthetics between Group I and Group II of the study participants (*n* = 50).

Variables	Group I (*n* = 25)	Group II (*n* = 25)	*p* value^∗^
Score ≥ 7.0 and score ≤ 8.0 *n* (%)	9.0 (36.0)	12.0 (48.0)	0.48
Score 9.0 *n* (%)	13.0 (52.0)	12.0 (48.0)	
Score 10.0 *n* (%)	3.0 (12.0)	1.0 (4.0)	

^∗^ *p* < 0.05 considered as significant using Fisher's exact test.

## Data Availability

The data used to support the findings are included within the article.
